# A QoS Scheme for a Congestion Core Network Based on Dissimilar QoS Structures in Smart-Phone Environments

**DOI:** 10.3390/s101110006

**Published:** 2010-11-09

**Authors:** Sung-Ryong Hong, Wonshik Na, Jang-Mook Kang

**Affiliations:** 1 Dept. of General Education, Namseoul University, 21 Maeju-ri, Seonghwan-eup, Seobuk-gu, Cheonan-city, Choongnam, 331-707, Korea; E-Mails: srh@nsu.ac.kr (S.-R.H.); winner@nsu.ac.kr (W.N.); 2 Electronic Commerce Research Institute, Dongguk University, 707 Seokjang-dong, Gyeongju, Gyeongsangbuk-do, 780-714, Korea

**Keywords:** smart-phone, traffic classification, Diffserv, QoS, multimedia content

## Abstract

This study suggests an approach to effective transmission of multimedia content in a rapidly changing Internet environment including smart-phones. Guaranteeing QoS in networks is currently an important research topic. When transmitting Assured Forwarding (AF) packets in a Multi-DiffServ network environment, network A may assign priority in an order AF1, AF2, AF3 and AF4; on the other hand, network B may reverse the order to a priority AF4, AF3, AF2 and AF1. In this case, the AF1 packets that received the best quality of service in network A will receive the lowest in network B, which may result in dropping of packets in network B and *vice versa*. This study suggests a way to guarantee QoS between hosts by minimizing the loss of AF packet class when one network transmits AF class packets to another network with differing principles. It is expected that QoS guarantees and their experimental value may be utilized as principles which can be applied to various mobile-web environments based on smart-phones.

## 1. Introduction

The Internet environment has changed and developed drastically, so few users now access it via modems, which were widely used a few years ago. As a result, the traditional Virtual Terminal (VT) environment has been replaced by the Graphic User Interface (GUI), and text-based simple HTML (Hypertext Markup Language) service has become a multimedia-based service. At an early stage, in an attempt to ensure QoS, the Internet Engineering Task Force (IETF) suggested the IntServ mechanism using the Resource ResServation Protocol (RSVP) Signaling Protocol, but this led to millions of resource reservations in the Core Router. The most critical problem is that all of the resource reservations must be configured again when the topology changes. This environment can seriously delay transmission of multimedia educational content and requires a complicated cost charge system because there is no limit to the number of classified services demanded by each flow. To solve the above problem, IETF suggested the Diffserv mechanism now in practical use. Diffserv classifies packets into three classes: EF (Expedited Forwarding), AF (Assured Forwarding), and DE (Default Forwarding). This study suggests a way of transmitting multimedia contents with the best quality, whether the network provides DiffServ or not [1]. To achieve this goal, the proposed method will rewrite the unused 2 bits of Differentiated Service Code Point (DSCP) value among the 8 DiffServ DSCP value bits.

## 2. Related Work

The Diffserv sets up DSCP values after classifying three EF, AF and DE service classes in edge routers. Then it transmits values to core routers that after receiving DSCP values, determine Per Hop Behavior (PHB). Since the DiffServ identifies the DSCP values by using 6 bits out of the 8 bits which are Type of Service (TOS) bytes in the IT Header, the network can provide differentiated services according to the packets [2]. When data comes from other networks, Multi-DiffServ first checks if it is transmitted through the proper route as contracted. Then, it allows packets to go to the core routers through the Routing Core and PHB. In this case, the DSCP values can be reconfigured or be transmitted without change. In the same DiffServ, proprietary DSCP values can be defined.

### 2.1. AF(Assured Forwarding) Class

**Figure 1.**
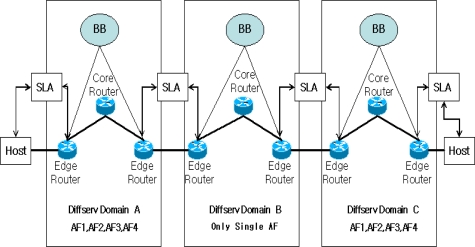
An Example of Multi-DiffServ Architecture.

The DiffServ has EF, AF, and DE classes of packets, of which the AF Class is separated divided into four classes (AF1, AF2, AF3, AF4), so in total six separate classes are provided. Due to the characteristics of the DiffServ network, AF1 given the highest priority in the AF class can drop to the lowest priority class when switching networks. A single network may apply a uniform policy, but it is not easy to apply the same policy in a multinetwork, given the different characteristics of Internet Service Providers (ISPs) [3,4]. As seen in Figure 1, if there are networks A, B and C and packets will be transmitted from A to B and C and networks A and C have the same AF packet priority order, but B simply provides AF, when a packet departing network A enters network B, the packet with AF1 value will lose its value and be provided with service as an AF packet.

Even though this packet enters network C through network B, the packet cannot receive AF1 level service since it has lost the AF1 value. Even if a packet can directly enter network C, there can be a problem, too. If network C has an AF packet conditioning process opposite to that in network A, a packet of the highest service in network A will receive the lowest service in network C.

### 2.2. Reconfiguration of DSCP in AF Class

**Figure 2.**
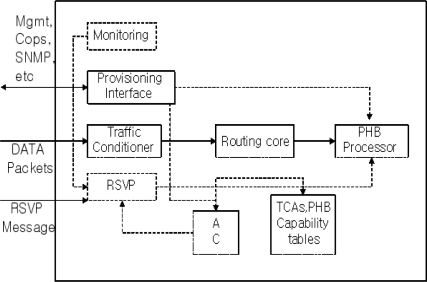
Mechanism of Diffserv Edge Router.

Multi-DiffServ service sets up a bilateral Service Level Agreement (SLA) with its neighbor DiffServ networks, and contains information on Service Type, Service Type Parameters and Service Restrictions [5]. As seen in Figure 2, the traffic conditioner in edge routers first checks the data from other networks. After examining if user traffic sent packets as contracted, the Multi-DiffServ sends packets over the routing core and PHB to the core routers. The Multi-DiffServ can reconfigure DSCP values or maintain the original conditions. The same DiffServ can use its defined proprietary DSCP values.

In an instance that a network receives an AF1 packet from a neighbor network and the current network does not define AF1, the network has the proprietary DSCP value of the existing DiffServ. Therefore, it will encode the DSCP value and send it to the core routers. Then it will perform PHB and succeed in transmitting the AF packet.

## 3. Suggested Method and Mathematical Confirmation

**Figure 3.**
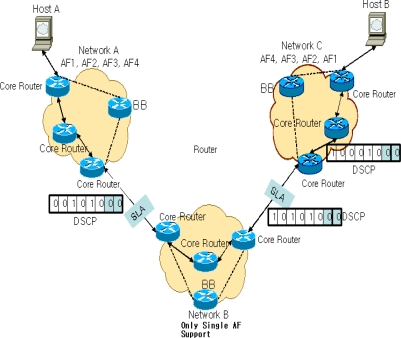
IP Datagram.

This paper suggests transmitting packets after coding DSCP at TC of edge routers by using the unused 2 bits. As seen in Figure 3, if Host A in the Multi-DiffServ network A transmits AF11 packet to Host B in network C, the configured DSCP value would be “0 0 1 0 1 0 0 0” and it passes the core routers in network A and reaches the edge routers which are in the middle of network A and network B.

Because networks A and B have an agreement on a single AF, packets from network A enter the edge routers in network B and the TC changes its DSCP value to a single AF value. If the single AF value is supposed to be 101010, the DSCP value would be “1 0 1 0 1 0 0 0 ” and passes over the core routers in network B to the routers at the edge of network C. Due to the SLA between networks B and C, the packets will be transmitted to network C. Then, the TC in network C recognizes the packets as AF, but the last two bits 00 tells that its drop preference is low. These packets will be randomly coded with the DSCP value that goes with AF low and sent to the core routers. If a low DSCP value is randomly allocated, the final value may or may not be the original AF11 value. The drop preference of AF packets, however, is low, and thus it has an advantage that the original service is guaranteed to a certain degree, even though the setting of the AF packet is different. Another advantage is that AF packets can be exchanged according to the class without other configuration.

**Figure 4.**
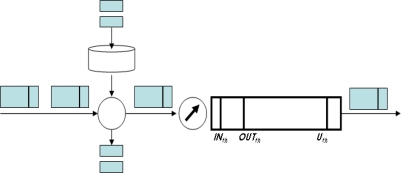
Structure of the Three-Phased Traffic Conditioner.

As shown in Figure 4, three PTC composites consist of Token Bucket phase, Probability Decision phase and Buffer Management phase. With sophisticated algorithms, each phase controls traffic coming into the DiffServ network by filtering packets from AS (Assured Services) users in order sequence.

At the Token Bucket phase, the token bucket measures traffic, and the packets will be re-marked from OUT to IN when an arbitrary user violates the contracted bandwidth. Equations 3, 4 and 5 show the Probability Decision phase. Probability means the opportunity that packets belonging to an arbitrary traffic flow *i* can enter the DiffServ network and is indicated as *WP* ( $p_{\mathrm{write}}^{i}$).

This paper assumes that edge routers ensures AS users’ DiffServ network and contracted bandwidth ( $r_{\mathrm{rev}}^{i}$). WP is decided by the contracted bandwidth ( $r_{\mathrm{rev}}^{i}$) and the amount of the token bucket measured traffic that reaches the edge routers. In order to calculate WP, it is necessary to define *rate ratio* ( $r_{\mathrm{rev}}^{i}/r_{\max}$), which is the ratio of the contracted bandwidth of each AS traffic to a router and is used as a basic probability in calculating WP. *r_max_* represents the maximum value among $r_{\mathrm{rev}}^{i}$ of the various AS traffics. AS traffic that reaches edge routers is accumulated for a defined time window. WP is calculated from the accumulated traffic( $\sum r_{\mathrm{rev}}^{i}r$) and *rate ratio*. Each TCP and UDP have different WP decision formulas.

First, the TCP WP decision formulae are shown in Equations 3, 4, and 5. Terms *α* and *β* are obtained from the difference between $r_{\mathrm{arr}}^{i}$ and $r_{\mathrm{rev}}^{i}$. If $r_{\mathrm{arr}}^{i}$ is smaller than $r_{\mathrm{rev}}^{i}$ for one time window, *α* is decided by the difference between the amount of the arrival traffic and the contracted bandwidth; if $r_{\mathrm{arr}}^{i}$ is bigger than $r_{\mathrm{rev}}^{i}$, *β* is decided from the amount of the arrival traffic exceeding the contracted bandwidth. On the basis of this concept and with the computer simulation, the formulae for *α* and *β* are deduced as follows:

(1) $$\alpha =\frac{r_{\mathrm{rev}}^{i}-r_{\mathrm{arr}}^{i}}{r_{\mathrm{rev}}^{i}}$$

(2) $$\beta =\frac{r_{\mathrm{arr}}^{i}-r_{\mathrm{rev}}^{i}}{r_{\mathrm{arr}}^{i}}\times \frac{r_{\mathrm{rev}}^{i}}{r_{\text{max}}}\times 0.5$$

It is a problem that TC0P traffic of narrow contracted bandwidth or small RTT overuses the network resources so that AS users who have comparatively wide bandwidth or large RTT fail to achieve the contracted service level:

(3) $$\mathrm{if}(r_{\mathrm{arr}}^{i}<r_{\mathrm{rev}}^{i}),\;\;p_{\mathrm{write}}^{i}=\frac{r_{\mathrm{rev}}^{i}}{r_{\text{max}}}+\alpha$$

(4) $$\mathrm{if}(r_{\mathrm{arr}}^{i}>r_{\mathrm{rev}}^{i}),\;\;p_{\mathrm{write}}^{i}=\frac{r_{\mathrm{rev}}^{i}}{r_{\text{max}}}-\beta$$

(5) $$\mathrm{if}(r_{\mathrm{arr}}^{i}>r_{\mathrm{rev}}^{i})\&(n\geq 2),\;p_{\mathrm{write}}^{i}=\left(\frac{r_{\mathrm{rev}}^{i}}{r_{\text{max}}}-\beta \right)\times {\left(\frac{r_{\mathrm{rev}}^{i}}{r_{\text{max}}}\right)}^{n}$$

## 4. Results and Analysis

**Figure 5.**
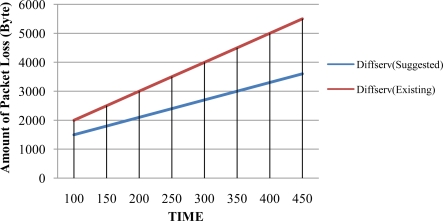
Comparison of the Amount of Packets Loss per Unit Time.

In order to test the performance of the suggested DiffServ method and the existing IEFT DiffServ method, this study used the ns2 simulator and adopted the topology of a smart-phone environment. Host A sends packets to Host B and passes over four routers. The test environment for the study assumes that the DiffServ network environment is settled and the suggested DiffServ module is on the Host. Constant bit rate (CBR) is used for Traffic, the packet size is 1,024 bytes, and Modified WRR Mode2 (MWRR2) Scheduling Algorithms are used. Figure 5 displays the amount of packets received by Host B from Host A per unit time thorough the DiffServ network or the smart-phone environment.

**Figure 6.**
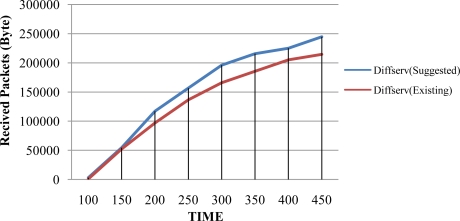
Comparison of the Received Packets per Unit Time.

As seen in Figure 5, the advantage of the suggested DiffServ is not discernably remarkable for the first 100 seconds because both networks have received almost same amount of packets during this period; however, the graph shows differences after 200 seconds and it is obvious that the suggested DiffServ received larger packets than the other after 400 seconds, which was the last test point. Sending a small amount of packets will not make a big difference. Therefore, the suggested method can produce more satisfactory results when sending a large amount of packets for a longer period of time. Figure 6 shows the comparative success rate of receiving packets between of the suggested DiffServ and the existing DiffServ when packet transmission is increased. The graph indicates that packets are transmitted from Host A to Host B and shows the amount of packets received at Host B.

## 5. Conclusions

When traffic congestion occurs in a DiffServ network, the network does not guarantee QoS in order to decrease the traffic. In the instances of DiffServ traffic congestion, it is necessary to transmit packets more effectively [6]. To cope with the congestion better than the existing DiffServ network does, this study suggests the following method: the host determines a DSCP value and sends it directly to the edge routers, so that these do not need to set up the DSCP value but only to make the core routers identify the PHB for the packets. Consequently, this method decreases traffic load on the edge routers and the workload to define DSCP values. The problem with this suggestion is that it is impossible for all the service providers to set the same DiffServ principles, therefore, EF or DE Class can be provided with a single service, but AF Class can be provided with four separate classes. This method can avoid the problem and produce a more efficient DiffServ network architecture. It is expected that QoS guarantees and their experimental values may be utilized as a principle which can be applied to various mobile-web environments based on smart-phones.
